# *MDGA2* homozygous loss-of-function variants cause developmental and epileptic encephalopathy

**DOI:** 10.1016/j.ajhg.2025.12.015

**Published:** 2026-01-21

**Authors:** Heba Morsy, Hyeonho Kim, Gyubin Jang, Maha S. Zaki, Mariasavina Severino, Ibrahim M. Abdelrazek, Haytham Hussien, Eleanor Self, Raidah Saleem Albaradie, Khadijah Bakur, Zahra Firoozfar, Stephanie Efthymiou, Mahmoud M. Noureldeen, Amira Nabil, Javeria Raza Alvi, Fateme Molavi, Shahryar Alavi, Reza Alibakhshi, Vehap Topcu, Hanifenur Mancilar, Eyyup Uctepe, Ahmet Yesilyurt, Hesham Aldhalaan, Ehab Salah Showki Tous, Bader Alhaddad, Hasnaa M. Elbendary, Annarita Scardamaglia, David Murphy, Vicente A. Yépez, Julien Gagneur, Tarek I. Omar, Marwa Abd Elmaksoud, Jana Vandrovocova, Ebtessam Abdalla, Mary M. Reilly, Tipu Sultan, Fowzan S. Alkuraya, Joseph G. Gleeson, Ji Won Um, Henry Houlden, Jaewon Ko, Reza Maroofian

**Affiliations:** 1 Department of Neuromuscular Diseases, UCL Institute of Neurology, University College London, Queen Square, London WC1N 3BG, UK; 2 Department of Human Genetics, Medical Research Institute, Alexandria University, Alexandria, Egypt; 3 Department of Brain Sciences, Daegu Gyeongbuk Institute of Science and Technology (DGIST), Daegu 42988, Korea; 4 Center for Synapse Diversity and Specificity, DGIST, Daegu 42988, Korea; 5 Clinical Genetics Department, Human Genetics and Genome Research Institute, National Research Centre, Cairo, Egypt; 6 Neuroradiology Unit, IRCCS Istituto Giannina Gaslini, 16147 Genoa, Italy; 7 Kuwait Hospital, Sabah Al-Salem, Block1, Kuwait; 8 King Fahd Specialist Hospital, Dammam, Saudi Arabia; 9 Lifera Omics, Riyadh 13519, Saudi Arabia; 10 Palindrome, Isfahan, Iran; 11 Department of Paediatrics, Faculty of Medicine, Beni-Suef University, Beni-Suef, Egypt; 12 Department of Paediatric Neurology, Institute of Child Health, Children’s Hospital Lahore, Lahore, Pakistan; 13 Dr. ALibakhshi Medical Genetics Laboratory, Kermanshah, Iran; 14 Department of Animal Biology, Faculty of Biological Sciences, Kharazmi University, Tehran, Iran; 15 Department of Neurodegenerative Diseases, UCL Queen Square Institute of Neurology, University College London, London, UK; 16 Department of Biochemistry, Kermanshah University of Medical Sciences, Kermanshah, Iran; 17 Acibadem Labgen Genetic Diagnosis Center, Istanbul, Türkiye; 18 Acibadem Maslak Hospital, Istanbul, Türkiye; 19 Neuroscience Centre of Excellence, KFSH&RC, Riyadh, Saudi Arabia; 20 Department of Clinical and Movement Neurosciences, UCL Queen Square Institute of Neurology, Queen Square, London WC1N 3BG, UK; 21 School of Computation, Information and Technology, Technical University of Munich, Munich, Germany; 22 Computational Health Center, Helmholtz Munich, Neuherberg, Germany; 23 Institute of Human Genetics, School of Medicine and Health, Technical University of Munich, Munich, Germany; 24 Neurology Unit, Department of Paediatrics, Faculty of Medicine, Alexandria University, Alexandria, Egypt; 25 Department of Translational Genomics, Genomic Medicine Centre of Excellence, King Faisal Specialist Hospital and Research Centre, Riyadh 11211, Saudi Arabia; 26 College of Medicine, Alfaisal University, Riyadh, Saudi Arabia; 27 Department of Neurosciences, University of California, San Diego, La Jolla CA 92093, USA

**Keywords:** MDGA2, neurodevelopmental disorder, epileptic encephalopathy, excitatory synapse, excitatory-inhibitory balance, synaptic suppression, neuroligin

## Abstract

*MDGA2* encodes a membrane-associated protein that is critical for regulating glutamatergic synapse development, modulating neuroligins (Nlgns), and maintaining excitatory-inhibitory synaptic balance. While MDGA2 functions have been extensively studied in murine and cellular models, its association with human developmental disorders has yet to be established. Through exome sequencing, we identified seven distinct homozygous loss-of-function variants in *MDGA2* in nine individuals from seven consanguineous families, all presenting with developmental and epileptic encephalopathy (DEE). Clinically, these individuals exhibited a consistent phenotype including infantile hypotonia, severe neurodevelopmental delay, intractable seizures, along with distinct dysmorphic features. Neuroimaging findings included delayed/incomplete myelination, early-onset brain atrophy, white-matter thinning, basal ganglia volume loss, and small hippocampi. Functional studies of three representative nonsense variants revealed impaired MDGA2 membrane trafficking, disrupted Nlgn1 interaction, and perturbed MDGA2-mediated excitatory synaptic functions in mammalian expression systems and cultured hippocampal neurons. Our findings support the involvement of *MDGA2* in a subtype of autosomal-recessive DEE. This not only underscores a loss-of-function pathogenic mechanism but also highlights the previously unrecognized role of MDGA2 in human synaptic development and regulation, significantly expanding our understanding of the genetic architecture of DEEs.

## Graphical abstract

**Figure:**
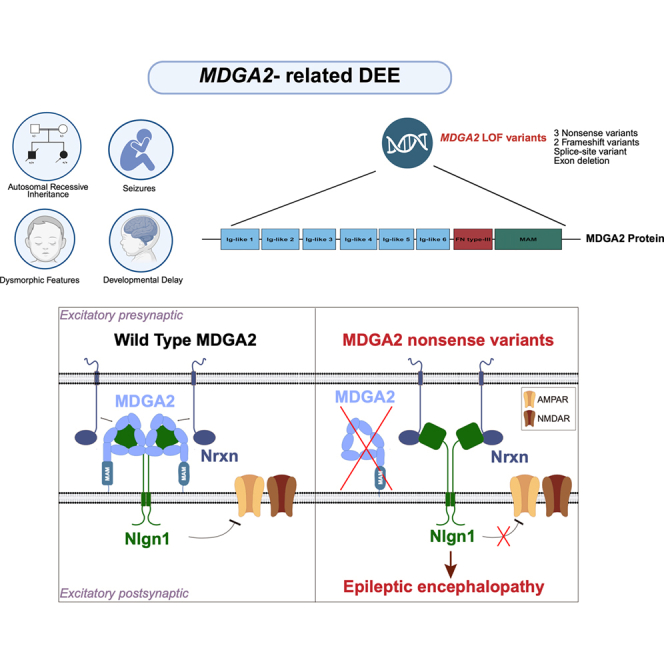

## Teaser

Morsy, Kim, and Jang et al. identify homozygous loss-of-function variants in *MDGA2* in individuals with autosomal-recessive developmental and epileptic encephalopathy (DEE). Functional data demonstrate impaired MDGA2 trafficking and disrupted MDGA2-dependent synaptic regulation, supporting its role in human neurodevelopment and expanding the genetic landscape of DEE.

## Main text

Developmental and epileptic encephalopathies (DEEs) are a group of severe neurodevelopmental disorders characterized by early-onset, intractable seizures and intellectual disability or developmental regression. These conditions have complex and heterogeneous etiology and usually result from genetic variants that interfere with normal brain development and function. Despite significant advances in the identification of the genetic causes involved in DEEs, many affected individuals remain without defined molecular defects and, hence, adequate genetic counseling. Establishing a genetic basis for these conditions considerably influences treatment strategies and clinical decision-making.^1^^,^^2^

In the present study, we identified 17 affected individuals, eight of whom had passed away. Exome sequencing was performed on nine affected individuals (six males, three females; aged 6 months to 17 years), revealing homozygous variants in MAM domain-containing glycosylphosphatidylinositol anchor 2 (*MDGA2* [MIM: 611128]). All nine individuals presented with common clinical features summarized in Table 1. Case reports and detailed clinical history are provided in supplemental information and Table S1. Video recordings are available for affected individuals from families 3, 4, and 5 (Videos S1, S2, S3, and S4).

**Table 1:** Clinical summary of individuals with homozygous DEE-linked *MDGA2* variants

**Family ID**	**F1**	**F2**	**F3**		**F4**	**F5**		**F6**	**F7**
**Individual ID**	**P1**	**P2**	**P3A**	**P3B**	**P4**	**P5A**	**P5B**	**P6**	**P7**
Genomic position (NC_000014.9)	g.46873446A>C	g.47217924_47218293del	g.47218196C>T		g.47061564G>A	g.46855148A>C		g.47096882del	g.47096882dup
cDNA (NM_001113498.3)	c.2739T>G	c.421-96_595 + 99del	c.421-1G>A		c.1210C>T	c.2759T>G		c.1172del	c.1172dup
Protein (NP_001106970.4)	p.(Tyr913Ter)	p.?	p.?		p.(Arg404Ter)	p.(Leu920Ter)		p.(Lys391SerfsTer7)	p.(Ser392ValfsTer25)
Ethnic background	Egyptian	Egyptian	Egyptian		East Asian	Saudi		Saudi	Iranian
Age at last FU	6 months	16 months	6 years 4 months	5 years 2 months	passed away at 2.5years	passed away at 4.5 years	17 years	passed away	12 years
Hypotonia in infancy	+	+	+	+	+	+	+	N/A	+
GDD	severe	severe	severe	severe	severe	severe	severe	N/A	severe
Psychomotor regression	yes (3 months)	no, since early life	yes (5 months)	yes (3 months)	yes (3 months)	no, since early life	no, since early life	N/A	no, since early life
Progressive disease	+	+	+	+	+	+	+	N/A	+
Sleep problems	+	+	+	–	+	+	+	N/A	–
Seizure type (age of onset)	GTCS, focal, myoclonic (3 months)	GTCS, atonic (50 days)	focal with secondary generalization tonic-clonic (5 months)	focal with secondary generalization tonic-clonic (2 months)	focal with secondary generalization tonic-clonic (2 months)	myoclonic, tonic, clonic, focal GTCS. (1 months)	epileptic spasm, tonic (2 months)	N/A	N/A (9 months)
Seizure treatment response	intractable	intractable	controlled	intractable	intractable	partial control	intractable	N/A	intractable
DTR	brisk	brisk	brisk	brisk	elicited	brisk	N/A	N/A	elicited
Muscle tone	normal	normal	hypotonia	hypotonia	normal	hypotonia	N/A	N/A	hypotonia
Muscle weakness	–	+	+	+	–	+	N/A	N/A	+
Abnormal mov	+	+	+	–	+	–	N/A	N/A	–
Dysmorphic features	+	+	+	+	+	+	+	N/A	+
Age at brain MRI	6 months	4 months	6 months	2 years	1 year	N/A	3 years	N/A	1 year
Brain atrophy	+	+	+	+	+	N/A	+	N/A	+
Delayed myelination	+	+	+	+	+	N/A	–	N/A	+
Small BG and hippocampi	+	+	+	+	+	N/A	+	N/A	+

BG, basal ganglia; DTR, deep-tendon reflexes; FU, follow-up; GDD, global developmental delay; GTCS, generalized tonic-clonic seizures; mov, movement; N/A, not available.

Video S1. Phenotype of individual P3A She has psychomotor delay; she can only sit few seconds. Note also abnormal sounds and involuntary movements.

Video S2. Phenotype of individual P3B He has psychomotor delay and shows abnormal sounds and involuntary movements. He is more affected than his sister (P3A).

Video S3. Phenotype of individual P4 He has developmental delay and strabismus

Video S4. Phenotype of individual 5B She has severe psychomotor delay and abnormal hand movements. Note dysmorphic facial features.

**Figure 1:**
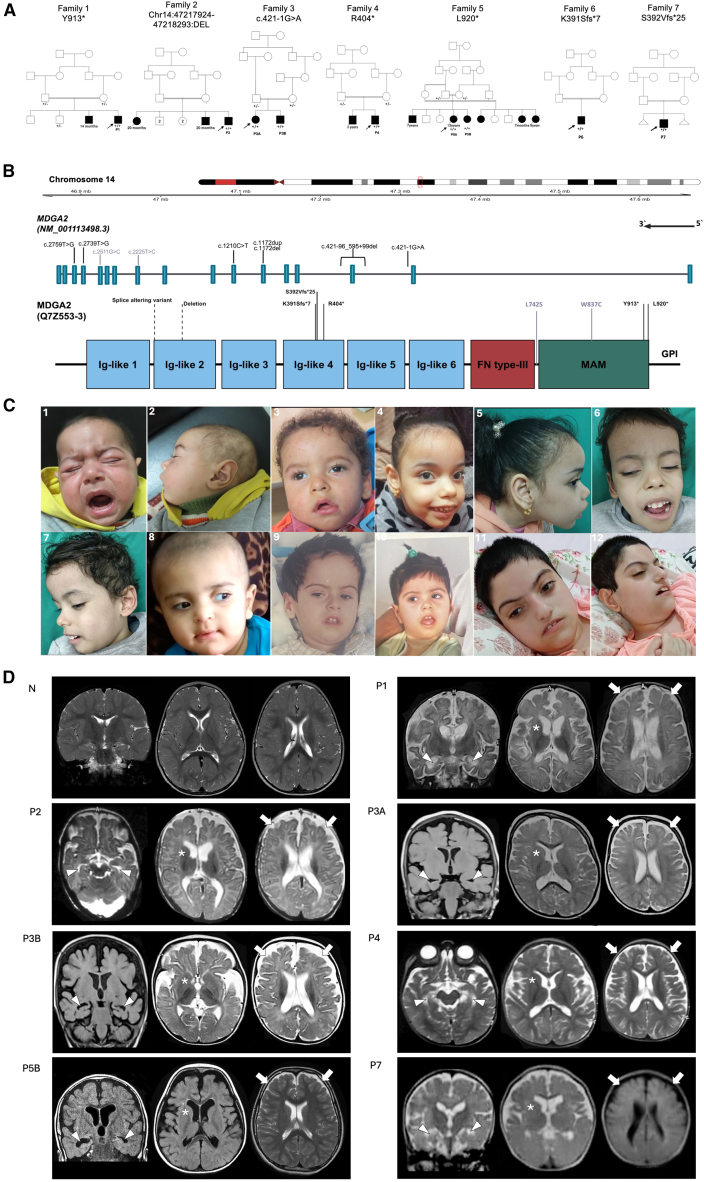
Family pedigrees, genetic variants, and dysmorphic and neuroradiological findings of individuals with DEE-linked *MDGA2* variants (A) Pedigrees of the seven unrelated families showing consanguinity and the identified *MDGA2* variants with genotypes indicated for available family members. (B) Schematic structure of *MDGA2* gene (top) and its encoded protein (bottom) highlighting variants identified in this study. Deletion and splice variants are represented in the MDGA2 protein by approximate dashed lines, as precise locations in protein are not known. Missense variants, which were not included in the main cohort due to inconclusive functional evidence, are shown in gray. (C) Dysmorphic facial features in seven affected individuals, in frontal and lateral views. P1 (C1 and C2) showed dolichocephaly, high anterior hairline, thin arched eyebrows, broad nasal bridge, bulbous nasal tip, tented upper lip, and large low-set ears with prominent antihelix. P2 (C3) showed high anterior hairline, arched eyebrows, long eyelashes, inner epicanthic fold, right upslanting palpebral fissure, strabismus, broad nasal bridge, bulbous nasal tip, and tented upper lip. P3A (C4 and C5) showed dolichocephaly, high anterior hairline, high prominent forehead, prominent maxilla, bluish sclera, strabismus, upslanting palpebral fissures, thin highly arched eyebrows with medial flare, broad nasal bridge, short philtrum, tented upper lip, and large low-set ears. P3B (C6 and C7) showed high anterior hairline, high forehead with intertemporal narrowing, mild hirsutism, strabismus, long eyelashes, inner epicanthic folds, thin arched eyebrows, short broad nasal bridge, tented upper lip, and large low-set ears. P4 (C8) showed high anterior hairline, high forehead with frontal bossing, bluish sclera, strabismus, upslanting palpebral fissure, broad nasal bridge, underdeveloped alae nasi, downturned corners of the mouth, and low-set posteriorly rotated ears. P5A (C9) showed biparietal bossing, high anterior hairline, high forehead with intertemporal narrowing, frontal bossing, thin high-arched eyebrows, right ptosis, strabismus, upslanting palpebral fissure, broad nasal bridge, underdeveloped alae nasi, short philtrum, thin tented upper lip, thin lower lip, and low-set ears. P5B (C10 at 5 months and C11, C12 at 17 years old) illustrates age-related progression and increased prominence of dysmorphic facial features. At 17 years old, P5B showed high anterior hairline, long face, narrow forehead, thin high-arched eyebrows, long eyelashes, upslanting palpebral fissure, short smooth philtrum, thin tented upper lip, thin lower lip, downturned corners of the mouth, and large low-set ears. (D) Neuroradiological features. Brain MRI of a normal control (N) at 13 months of age, of subject P1 at 6 months, subject P2 at 4 months, subject P3A at 2 years, subject P3B at 4 months, subject P4 at 1 year, P5B at 3 years, and P7 at 1 year. All but one subjects show delayed or incomplete myelination (P5B has normal white-matter signal at 3 years). Moderate to severe white-matter volume loss with consequent ventricular dilatation and increased cerebral subarachnoid spaces (thick arrows) is observed in all individuals. Reduced volume of the basal ganglia (asterisks), particularly in the caudate nuclei and thalami, and of the hippocampi (arrowheads) is also noted.

All individuals presented with DEE and global developmental delay. The ethnicity of all families was Middle Eastern (Egyptian, Saudi, and Iranian) except family 4, which originated from Southeast Asia. All families reported consanguinity, and family histories consistently revealed similarly affected siblings and relatives, often with early mortality (Figure 1A). Although a detailed clinical examination was not available for individual P6, as he passed away early in life, we included him in the cohort due to the presence of a frameshift variant consistent with the proposed loss-of-function mechanism, along with core clinical features of the disorder, including severe DEE and global developmental delay. We focus the description below on the eight individuals from the other six families.

All affected individuals exhibited severe motor and language impairment. Most did not attain key developmental milestones; none achieved independent ambulation, and only two (P3A and P7) were able to sit without support. Severe infantile hypotonia was universally observed. Psychomotor regression was reported in four individuals, typically beginning within the first months of life. The overall disease course was progressive in all individuals. Behavioral symptoms were noted in four individuals, with sleep disturbances reported in all but two individuals (P3B and P7).

Seizures occurred early in life in all affected individuals with a broad spectrum of seizure types, including generalized tonic-clonic, focal, myoclonic, tonic, epileptic spasms, and atonic seizures. Recurrent status epilepticus was documented in three individuals (P3A, P3B, and P4). Electroencephalogram (EEG) studies consistently demonstrated generalized slowing and encephalopathy in all seven individuals with available data. Treatment response to anti-epileptic drugs (AEDs) was generally poor; however, P4 and P5B achieved partial seizure control following a ketogenic diet. Deep-tendon reflexes were brisk in six individuals, and muscle tone abnormality was noted in P3A, P3B, P5A, and P7, who exhibited hypotonia and muscle weakness. There was no evidence of muscle atrophy or sensory/autonomic abnormality. Abnormal movements were observed in all individuals except P3B, P5A, and P7.

All affected individuals exhibited craniofacial abnormalities, with frontal prominence observed in almost all individuals. A high anterior hairline and high forehead were consistent findings across most individuals (6/8). Dolichocephaly was observed in P1 and P3A, while intertemporal narrowing was shared by P1, P3B, P5A, and P7. Periorbital features included thin, highly arched eyebrows, noted in nearly all individuals (7/8), occasionally with a medial flare (P3A and P7). Strabismus was common (6/8), and upslanting palpebral fissures were observed in four individuals. Long eyelashes were observed in P2, P3B, and P5B. The nasal region was consistently affected, with all individuals displaying a broad nasal bridge. Additional features such as underdeveloped nasal alae and bulbous nasal tip were variably present. Orofacial abnormalities were prominent, with a tented upper lip observed in almost all individuals (7/8). In the affected siblings in family 5, this was accompanied by a thin lower lip. Downturned corners of the mouth were noted in P4 and P5B. Most individuals had large, low-set ears (dysmorphic facial features are shown in Figure 1C). Progressive age-related evolution of facial characteristics was evident, with features becoming more pronounced over time, as illustrated in P5A (Figure 1C) and the two affected siblings from family 3 (Figure S1).

Brain magnetic resonance imaging (MRI) studies were available for review in seven individuals and were performed at an average age of 1.2 years (range, 6 months to 3 years). Delayed or incomplete myelination was observed in six of the seven individuals. All individuals with available neuroimaging exhibited early-onset brain atrophy with thinning of the white matter, basal ganglia volume loss, and small hippocampi (Figure 1D). In one subject (P1), the first brain MRI performed at 3 months of age revealed widespread T2 signal alterations with restricted diffusion in the cerebral cortico-subcortical and deep white-matter regions; these features rapidly evolved into severe brain atrophy on the follow-up MRI performed at 6 months of age.

Ethical approvals were obtained in each recruiting center by the appropriate institutional review board (IRB), and all participants’ parents provided informed consent to participate in the research study. Proband-only whole-exome sequencing (WES) identified seven homozygous *MDGA2* variants in nine affected individuals. All identified variants were absent from gnomAD v3^3^ and were located within a region of homozygosity (ROH) (Tables S2, S3, S4, S5, S6, S7, S8, S9, and S10; Figure S2). Segregation of single-nucleotide variants (SNVs) was confirmed by Sanger sequencing in available family members (Figure S3).

The homozygous DEE-linked *MDGA2* variants (GenBank: NM_001113498.3 and NP_001106970.4) identified include three nonsense variants (p.Tyr913Ter [c.2739T>G] in P1, p.Arg404Ter [c.1210T>C] in P4, and p.Leu920Ter [c.2759T>G] in P5A and P5B); one splice variant (c.421-1G>A in P3A and P3B); two frameshift variants (p.Lys391SerfsTer7 [c.1172del] in P6 and p.Ser392ValfsTer25 [c.1172dup] in P7); and a homozygous deletion of exon 3 (c.421-96_595+99del) in P2 (Figures 1B, S4, S5, and S6). All identified variants are predicted to result in loss-of-function (LoF), consistent with the intolerance of *MDGA2* to LoF.^3^ The three nonsense variants are not in the last or penultimate exon of *MDGA2*, suggesting that mutated mRNA product could have increased susceptibility to nonsense-mediated mRNA decay (NMD). The splice variant identified in family 3 has a SpliceAI^4^ acceptor loss delta score (DS_AL) of 0.99 and is predicted to cause loss of the acceptor splice site, likely resulting in aberrant splicing and an LoF effect. In addition, testing the splicing effect of this variant using AbSplice^5^ showed a score of 0.054 (slightly above the pathogenic cutoff of 0.05) in multiple brain-tissue subtypes. The 370-bp homozygous deletion identified in P2 (g.47217924_47218293del [GenBank: NC_000014.9]) contains all of exon 3 and is predicted to lead to a frameshift, with the resulting transcript likely to undergo NMD. The two frameshift variants affecting the same nucleotide position (c.1172del and c.1172dup) result in a premature stop codon, which is predicted to produce a truncated MDGA2 protein or trigger NMD. The seven *MDGA2* variants reported herein might cause pathological truncation of MDGA2 protein, decrease *MDGA2* mRNA stability, and/or increase its degradation by NMD, as reported in other LoF-mediated genetic diseases.^6^

In addition, we evaluated two affected individuals carrying homozygous missense variants in *MDGA2*; proband M1 (c.2511G>C [p.Trp837Cys]) and proband M2 (c.2225T>C [p.Leu742Ser). M1 presented with global developmental delay, severe speech delay, intellectual disability, and refractory epilepsy. Brain MRI revealed delayed myelination. M2 showed mild global developmental delay, speech delay, and cerebral atrophy with reduced white-matter volume on brain MRI. Although functional studies were performed, the results were inconclusive, and these individuals were therefore not included in the primary cohort. Detailed clinical and genetic information for these individuals is provided in supplemental notes, Figure S7, and Table S11.

*MDGA2* is an evolutionarily conserved gene on chromosome 14q21.3. MDGA2, together with its paralog MDGA1, belongs to the neuronal glycosylphosphatidylinositol (GPI)-anchored proteins. Both MDGA paralogs are membrane-associated proteins that contain six tandem immunoglobulin (Ig)-like domains, a fibronectin-like region, a single meprin, A-5 protein, a receptor protein-tyrosine phosphatase mu (MAM) domain, and a C-terminal GPI anchor. They have been highlighted as key suppressive factors that tune the balanced activity of neural circuits. MDGA proteins expression is restricted to the central nervous system (CNS), begins early in development, and continues throughout adulthood.^7^^,^^8^^,^^9^^,^^10^^,^^11^^,^^12^

Prior studies established that MDGA2 negatively modulates glutamatergic synapses via distinct extracellular mechanisms. Studies using knockout (KO) mice showed that dysregulation of MDGA proteins could be associated with a subset of neuropsychiatric disorders, such as autism spectrum disorders (ASDs) and schizophrenia. It has been shown that mutation of *MDGA2* elevates excitatory transmission, and MDGA2 modulates neuroligin-1 (Nlgn1) interaction with neurexins and suppresses excitatory synapse development.^9^^,^^13^^,^^14^^,^^15^^,^^16^^,^^17^ To explore the effects of *MDGA2* variants on protein function, we tested three representative MDGA2 nonsense variants (Tyr844Ter [Y844∗], Arg335Ter [R335∗], and Leu851Ter [L851∗]) and two MDGA2 missense variants (Leu673Ser [L673S] and Trp768Cys [W768C]). Functional studies used isoform Q7Z553-1 for technical reasons (Figures S8A and S9A). The Tyr844Ter (Y844∗) nonsense variant corresponds to the variant identified in individual P1 (Tyr913Ter [Y913∗]) in the Q7Z553-3 isoform. Notably, these residues are evolutionarily conserved across species, hinting at their possible functional significance (Figure S9B). We assessed the impact of three nonsense MDGA2 variants using a mammalian cDNA expression vector but could not express cDNAs for the splice or deletion variants.

We first examined the expression levels and intracellular transport properties of the MDGA2 variants upon expression in human embryonic kidney 293T (HEK293T) cells. HEK293T cells were transfected with vectors encoding hemagglutinin (HA)-tagged full-length MDGA2 wild-type (WT) or variants. Immunoblotting of cell lysates showed that the total protein expression levels of the MDGA2 Leu673Ser, Trp768Cys, Tyr844Ter, and Leu851Ter were comparable to those of MDGA2 WT, whereas the R335^∗^ variant was not (Figure S8B). All three MDGA2 nonsense variants were expected to yield truncated proteins because they lacked membrane-anchored GPI sequence. However, although expressed Tyr844Ter and Leu851Ter migrated to slightly lower positions on sodium dodecyl sulfate polyacrylamide gel electrophoresis (SDS-PAGE) analyses and were not secreted, the Arg335Ter variant yielded a truncated protein that was ∼40 kDa on SDS-PAGE analyses and was prominently secreted (Figure S8B). We next examined the surface and intracellular protein levels of WT and the variant MDGA2 proteins in HEK293T cells (Figures S8C and S8D). The two MDGA2 missense variants exhibited surface expression levels comparable to that of MDGA2 WT; in contrast, none of the tested MDGA2 nonsense variants displayed detectable surface expressions in HEK293T cells, possibly reflecting their complete entrapment in an intracellular compartment.

To determine whether these MDGA2 variants would alter the interactions with Nlgn1, an extracellular ligand of MDGA2, we assayed the cell-surface binding of recombinant Ig-fusion proteins of Nlgn1 (Ig-Nlgn1) or IgC alone (negative control) with HEK293T cells expressing HA-tagged MDGA2 variants (Figures S8E and S8F). Our results revealed that the IgC-Nlgn1 proteins robustly bound to HEK293T cells expressing MDGA2 WT and the two MDGA2 missense variants but not the surface transport-defective MDGA2 variants. IgC did not bind to any tested MDGA2 variant. Overall, our results suggest that the tested MDGA2 nonsense variants lack the surface-trafficking and ligand-binding activities exhibited by MDGA2 WT.

**Figure 2:**
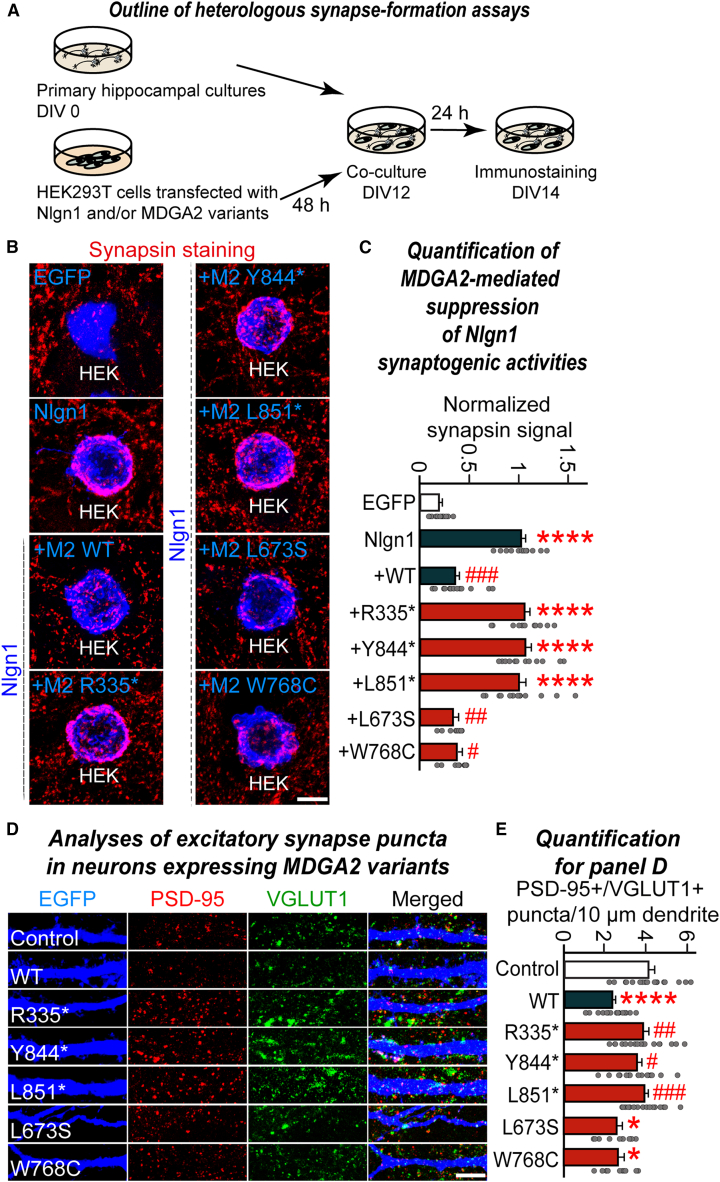
Nonsense *MDGA2* variants inactivate the anti-synaptogenic activity of MDGA2 WT and disrupt the negative regulation of excitatory synapse numbers in cultured hippocampal neurons (A) Overview of heterologous synapse-formation assays performed in the current study. (B and C) Effects of MDGA2 WT or its variants on Nlgn1-mediated synaptogenic activities. HEK293T cells expressing Nlgn1 were cocultured with hippocampal neurons without or with coexpression of the indicated MDGA2 variant. Representative images (B) of cocultures immunostained with antibodies to EGFP or HA (blue) and synapsin I (red). Quantitation (C) of heterologous synapse-formation assay results, as determined by calculating the signal ratio of synapsin I to EGFP/HA. Data are presented as means ± SEMs: ^∗∗∗∗^*p* < 0.0001 (vs. Control), ^#^*p* < 0.05, ^##^*p* < 0.01, ^###^*p* < 0.001 (vs. Nlgn1); nonparametric Kruskal-Wallis test with Dunn’s *post hoc* test; *n* = 12–19 cells/group. Scale bar, 10 μm (applies to all images). (D and E) Representative images (D) and summary graphs (E) showing the density of excitatory synaptic puncta in cultured hippocampal neurons transfected at DIV7 with the construct expressing the full-length MDGA2 or the indicated MDGA2 variant and immunostained at DIV14 with antibodies to VGLUT1, PSD-95, and EGFP. Data are presented as means ± SEMs: ^∗^*p* < 0.05, ^∗∗∗∗^*p* < 0.0001 (vs. Control), ^#^*p* < 0.05, ^##^*p* < 0.01, ^###^*p* < 0.001 (vs. WT); nonparametric Kruskal-Wallis test with Dunn’s *post hoc* test; *n* = 11–17 images/group.

MDGA2 was previously shown to inhibit the Nlgn1-mediated synaptogenic activity in driving presynaptic assembly in heterologous synapse-formation assays, where Nlgn1 expressed in heterologous cells recruits presynaptic components in axons of cocultured neurons.^12^ To test whether MDGA2 variants affected the ability of MDGA2 WT to inhibit Nlgn1-induced presynaptic differentiation-inducing activity, we performed heterologous synapse-formation assays using HEK293T cells expressing EGFP alone (negative control), expressing Nlgn1 alone, or coexpressing Nlgn1 with the indicated MDGA2 variants and applied anti-synapsin antibodies to label presynaptic sites (Figure 2A). Nlgn1 expressed in HEK293T cells strongly recruited presynaptic synapsin puncta in axons of cocultured neurons, and coexpression of MDGA2 WT or the indicated MDGA2 missense variants effectively decreased Nlgn1-induced presynaptic differentiation (Figures 2B and 2C). In contrast, none of the MDGA2 nonsense variants inhibited Nlgn1-mediated synaptogenic activity (Figures 2B and 2C). These observations are consistent with the above-described findings that all three nonsense MDGA2 variants lacked proper surface transport and ligand-binding activities.

We next examined whether the MDGA2 variants influenced the ability of MDGA2 WT to suppress the number of excitatory synapses in cultured hippocampal neurons. To this end, we cotransfected cells with expression vectors encoding EGFP and the various HA-tagged MDGA2 constructs at DIV7, and we immunostained the transfected neurons with antibodies to VGLUT1 (a marker for excitatory presynaptic terminal), PSD-95 (a marker for excitatory postsynaptic density), and EGFP (to visualize the transfected neurons) at DIV14. The density of excitatory synaptic puncta immunoreactive to both VGLUT1 and PSD-95 was significantly decreased in MDGA2 WT-expressing neurons (Figures 2D and 2E), in line with our recent report.^13^ Moreover, overexpression of the MDGA2 missense variants also suppressed the VGLUT1^+^PSD-95^+^ excitatory synaptic puncta (Figures 2D and 2E). In contrast, overexpression of all three MDGA2 nonsense variants failed to alter the density of excitatory synaptic puncta, in agreement with our results showing that these variants have deficits in surface transport and synaptogenic activity. As expected, all three MDGA2 nonsense variants exhibited impaired dendritic trafficking and reduced the HA immunofluorescence signals in the somatic compartment (Figure S10). Thus, our immunocytochemical analyses indicate that all tested MDGA2 nonsense variants act as LoF variants.

**Figure 3:**
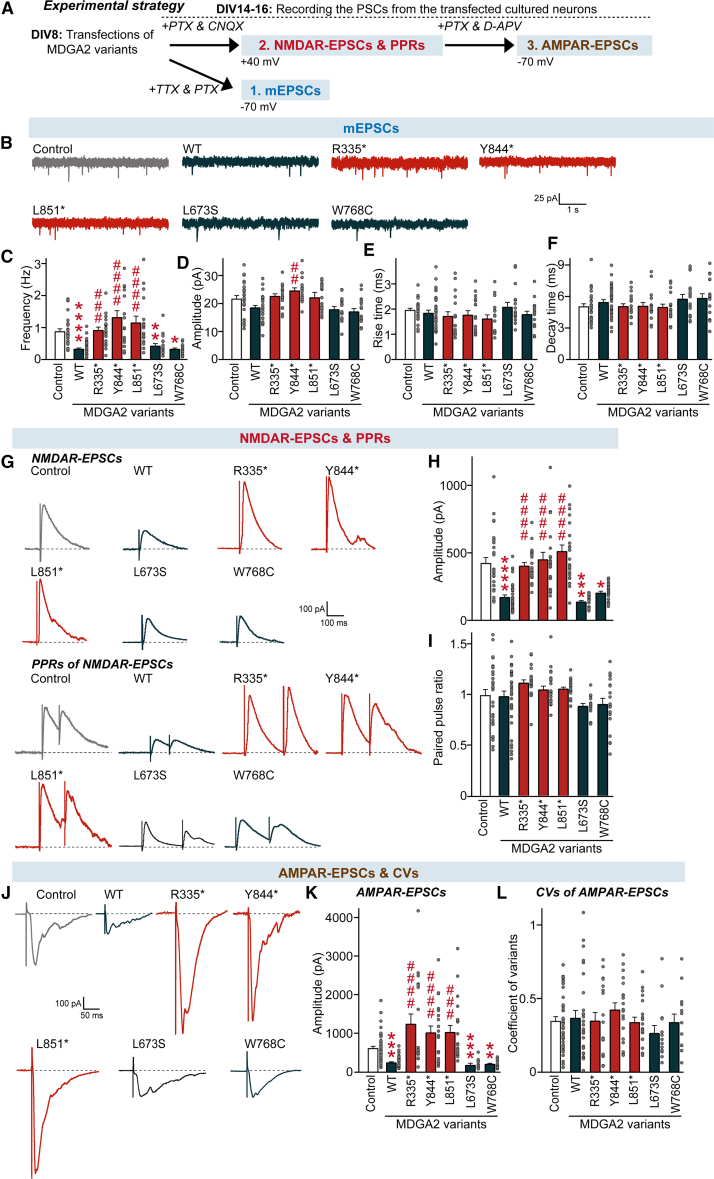
Nonsense *MDGA2* variants impair synapse suppression, disrupting the suppression of basal synaptic transmission and synaptic strength at excitatory synapses in cultured hippocampal neurons (A) Experimental strategy for recording miniature excitatory postsynaptic currents (mEPSCs), evoked AMPAR-mediated EPSCs (AMPAR-EPSCs), and evoked NMDAR-mediated EPSCs (NMDAR-EPSCs) from cultured hippocampal neurons transfected with the indicated MDGA2 variants. (B–F) Representative mEPSC traces (B) and quantification of the frequency (C), amplitude (D), rise time (E), and decay time (F) of mEPSCs. Data are presented as means ± SEMs (control, *n* = 26; WT, *n* = 31; R335^∗^, *n* = 18; Y844^∗^, *n* = 16; L851^∗^, *n* = 17; L742S, *n* = 16; W837C, *n* = 17. ^∗^*p* < 0.05, ^∗∗^*p* < 0.01, ^∗∗∗∗^*p* < 0.0001 (vs. Control), ^##^*p* < 0.01, ^###^*p* < 0.001, ^####^*p* < 0.0001 (vs. WT); nonparametric Kruskal-Wallis test with Dunn’s *post hoc* test). (G–I) Representative traces for evoked NMDAR-EPSCs and paired-pulse ratios (PPRs) of NMDAR-EPSCs (G) and quantification of the amplitude (H) and PPR (I) of evoked NMDAR-EPSCs. Data are presented as means ± SEMs (control, *n* = 28; WT, *n* = 33; R335^∗^, *n* = 21; Y844^∗^, *n* = 21; L851^∗^, *n* = 22; L742S, *n* = 14; W837C, *n* = 19; ^∗^*p* < 0.05, ^∗∗∗^*p* < 0.001, ^∗∗∗∗^*p* < 0.0001 (vs. Control), ^####^*p* < 0.0001 (vs. WT); nonparametric Kruskal-Wallis test with Dunn’s *post hoc* test). (J–L) Representative evoked AMPAR-EPSC traces (J) and quantification of amplitude (K) and coefficient of variation (CV) of AMPAR-EPSCs. (L) Data are presented as means ± SEMs (control, *n* = 39; WT, *n* = 28; R335^∗^, *n* = 18; Y844^∗^, *n* = 19; L851^∗^, *n* = 19; L742S, *n* = 14; W837C, *n* = 13; ^∗∗^*p* < 0.01, ^∗∗∗^*p* < 0.001 (vs. Control), ^###^*p* < 0.001, ^####^*p* < 0.0001 (vs. WT); nonparametric Kruskal-Wallis test with Dunn’s *post hoc* test).

To corroborate these results, we measured miniature excitatory postsynaptic currents (mEPSCs) in cultured hippocampal neurons using whole-cell electrophysiological recordings (Figure 3A). We found that the frequency, but not amplitude, of mEPSCs, was markedly decreased in cultured hippocampal neurons expressing MDGA2 WT or MDGA2 missense variant (Leu673Ser or Trp768Cys) (Figures 3B–3F), which was in line with our previous results.^13^ Overexpression of each of the nonsense MDGA2 variants failed to alter excitatory synaptic transmission (Figures 3B–3F). We next monitored evoked excitatory postsynaptic currents (EPSCs) by measuring AMPA (α-amino-3-hydroxy-5-methyl-4-isoxazolepropionic acid) receptor and NMDA (N-methyl-D-aspartate) receptor-mediated EPSCs (AMPAR-EPSCs and NMDAR-EPSCs, respectively) at holding potentials of −70 and +40 mV, respectively, in the presence of external Mg^2+^ (Figure 3A).

Overexpression of MDGA2 WT decreased the amplitude of both AMPAR-EPSCs and NMDAR-EPSCs, as previously reported.^13^ However, overexpression of any MDGA2 nonsense variant did not alter the amplitude of AMPAR-EPSCs or NMDAR-EPSCs (Figures 3G–3L). These results collectively reinforce that all three MDGA2 nonsense variants, but not the two MDGA2 missense variants, exhibit perturbation of MDGA2-mediated synaptic functions.

Altogether, we describe autosomal-recessive DEE syndrome caused by homozygous LoF variants in *MDGA2*, identified in nine individuals from seven consanguineous families. All individuals exhibited severe developmental delay, intractable seizures, and age-progressive dysmorphic facial features (high-arched eyebrows; broad nasal ridge; tented upper lip; and large, low-set ears). Notably, the condition exhibits high lethality, with many affected individuals dying in early infancy. In mouse models, constitutive MDGA2 deletion proved to be perinatally lethal, underscoring its critical role in normal brain development.^17^ Neuroimaging revealed nonspecific but consistent findings of early-onset brain atrophy, with volume reduction of the basal ganglia and hippocampi and delayed myelination, similar to findings in Syntaxin-binding protein 1 (*STXBP1* [MIM: 602926]) and Ferric chelate reductase 1-like (*FRRS1L* [MIM: 604574])-related DEEs,^18^ marking *MDGA2* as a new player in synaptic encephalopathies.

MDGA2, a synaptic regulator, suppresses density, transmission, and strength of glutamatergic synapses, including both NMDAR- and AMPAR-mediated postsynaptic responses.^19^ It exerts these effects by modulating NLGN1 through its immunoglobulin-like and MAM domains, thereby inhibiting the NLGN1-neurexin (NRXN) interactions critical for excitatory synapse formation.^13^ Both neuroligin-1 (*NLGN1* [MIM: 600568]) and neurexin I (*NRXN1* [MIM: 600565]) variants have been intimately linked to neurodevelopmental disorders.^20^^,^^21^ Our functional studies demonstrate that homozygous *MDGA2* nonsense variants affecting these key domains abolish MDGA2’s surface-trafficking and Nlgn1-binding activity, leading to unchecked excitatory synapse formation. This synaptic dysregulation is predicted to disrupt the cortical excitation-inhibition balance, a known driver of epileptogenesis shared with other DEEs.^22^ Furthermore, the progressive brain atrophy and delayed myelination observed on MRI suggest that *MDGA2* LoF exacerbates neuronal loss, potentially amplifying seizure severity.

Recent studies have implicated MAM domain-containing glycosylphosphatidylinositol anchor 1 (MDGA1 [MIM: 609626]), a paralog of MDGA2, in neurodevelopmental disorders, including ASDs. For example, missense variants in *MDGA1* reportedly impair CNS development and disrupt Nlgn2-mediated inhibitory synapse formation, altering synaptic balance.^14^^,^^23^ These findings and our present results highlight the broader role of the MDGA family in regulating synaptic organization and maintaining the excitation-inhibition balance.

Beyond seizures, MDGA2 dysfunction contributes to broader neurodevelopmental impairments in murine models, where *Mdga2*^+/−^ mouse haploinsufficiency causes delayed motor development, reduced ultrasonic vocalizations, and autism-like behaviors, including stereotypies, impaired social interactions, and memory deficits,^17^ paralleling the severe psychomotor delay and behavioral symptoms in our cohort. Additionally, the principal synaptic target for MDGA2, Nlgn1, maintains synchronous cortical activity during wakefulness and sleep,^21^ suggesting that disrupted MDGA2-Nlgn1 interactions may underlie the sleep disturbances consistently reported in our individuals.

Similar to other DEEs caused by defects in synaptic genes such as *STXBP1* and Cyclin-dependent kinase-like 5 (*CDKL5* [MIM: 300203]),^18^^,^^24^ which present with brain atrophy, delayed myelination, and craniofacial features, *MDGA2* LoF may also contribute to extra-synaptic abnormalities. These parallels suggest that synaptic dysfunction can extend to broader neurodevelopmental disturbances, potentially through impaired neuronal survival, disrupted axon-glia interactions, and/or additional developmental functions of synaptic proteins. These findings establish *MDGA2* LoF as a cause of DEE, with its synaptic defects directly contributing to the intractable seizure phenotype and broader neurodevelopmental impairments.

Recent work demonstrated that inhibition of TrkB activity or blockade of AMPAR signaling can attenuate social deficits in *Mdga2* heterozygous KO mice.^25^ This suggests potential therapeutic targets for *MDGA2*-related disorders that warrant further translational investigation. The partial seizure control achieved with the ketogenic diet in individuals P4 and P5B is also noteworthy. This dietary intervention is known to modulate neurotransmitter function^26^ and may help restore the disrupted excitation. Possibilities await validation in clinical trials, but the foundational work underscores the potential for mechanism-based therapeutic strategies and highlights the importance of understanding the underlying pathogenic mechanisms.

Here, we tested three representative nonsense variants and two missense variants. The identified splice, frameshift, and deletion variants are predicted to cause LoF via NMD or protein truncation. Their pathogenicity is consistent with the high LoF intolerance seen for *MDGA2* (pLI = 1). Notably, *MDGA2* also shows evidence of missense constraint, with a missense *Z* score of 2.03, indicating relative intolerance to amino acid-altering variation.^3^ Future studies investigating the pathogenicity of missense variants are worthwhile to examine whether these changes dysregulate the *MDGA2*-mediated suppression of glutamatergic synapses.

Although *MDGA2*’s roles have been explored in murine and cellular models,^13^^,^^16^^,^^17^ our present study establishes its essential contribution to human neurodevelopmental disorders. Nevertheless, our functional validation was limited to *in vitro* models, due to very low expression of MDGA2 in blood and skin based on Genotype-Tissue Expression (GTEx) consortium data,^27^ rendering studying *MDGA2*-related DEE variants in peripheral individual-derived samples challenging. This is further elucidated by RNA-sequencing data from blood and fibroblasts in the Solve-RD cohort, where MDGA2 expression was too low to be properly modeled (Figure S11). In contrast, brain-specific gene expression data show enrichment of MDGA2 in excitatory and inhibitory neurons and oligodendrocyte precursor cells, suggesting that it has cell-type-specific roles in the CNS.^28^ In addition, given that MDGA2 is also expressed in astrocytes, which plays an essential role in regulating the glutamatergic synapse,^29^ it would be interesting to apply systematic functional analyses to determine whether any aspects of astrocytic functions are altered. Future studies using individual-derived induced pluripotent stem cell (iPSC) neurons are highly recommended to confirm MDGA2’s role in human synaptic dysfunction, complementing our *in vitro* findings in mouse cultured neurons.

In conclusion, we establish *MDGA2* as a DEE gene, linking its LoF variants to a severe neurodevelopmental syndrome, through integrated clinical, genetic, and functional analyses. This study expands the genetic etiology of DEEs, holds promise for improving individuals’ outcomes with early diagnosis and genetic counseling, and lays the basis for targeted therapies, advancing our understanding of MDGA2 synaptic dysfunction in DEEs.

## Data and code availability

• The genomic data supporting the findings of this study consist of individual-level variant information and cannot be deposited in a public repository due to restrictions imposed by IRB approvals and participant consent agreements across participating centers.
• De-identified variant-level data for all *MDGA2* findings reported in this manuscript are provided within the article and its supplemental information.
• Additional population-genetic summary data underlying this study are available from the corresponding authors upon reasonable request, subject to approval by the relevant institutional review boards and in accordance with participant consent.
• All variants reported in this study will be submitted to ClinVar and will be publicly available upon publication of this manuscript.
• No custom code was generated for this study.

## Acknowledgments

We are grateful for the important support from participants and their families, our UK and international collaborators, and the SYNAPS Study Group. We acknowledge with gratitude the support provided by the Nile of Hope Hospital for Congenital Anomalies, Alexandria, Egypt. We are grateful to Jinha Kim (DGIST, Korea) for technical assistance. H.M. was supported by the 10.13039/100010269Wellcome Trust grant 220906/Z/20/Z and UCL Global Engagement Fund scheme 2023. H.K. was supported by the 10.13039/501100003725National Research Foundation of Korea (NRF) funded by the Ministry of Science and ICT (RS-2024-00339642). J.W.U. was supported by the NRF funded by the Ministry of Science and ICT (RS-2023-NR076948). J.K. was supported by the NRF funded by the Ministry of Science and ICT (RS-2022-NR070708). M.S.Z. was funded by GERF-STDF (33650, STDF, Egypt). H. Houlden was funded by the 10.13039/100010269Wellcome Trust, MRC, 10.13039/100013128MSA Trust, National Institute for Health Research University College London Hospitals Biomedical Research Centre (NIHR-BRC), 10.13039/100000864Michael J. Fox Foundation (MJFF), Fidelity Trust, 10.13039/501100000833Rosetrees Trust, 10.13039/100019709Dolby Family Fund, 10.13039/501100002283Alzheimer’s Research UK (ARUK), 10.13039/100018889MSA Coalition, Parkinson Disease Society, 10.13039/100013301Parkinson's Foundation, 10.13039/501100000627Guarantors of Brain, 10.13039/501100022099Cerebral Palsy Alliance, 10.13039/100002108FARA, 10.13039/501100011963EAN, Victoria Brain Bank, NIH NeuroBioBank, Queen Square BrainBank, and MRC Brainbank Network. V.A.Y. and J.G. were funded by the 10.13039/501100001659Deutsche Forschungsgemeinschaft (DFG [German Research Foundation]) via the project NFDI 1/1 “GHGA - German Human Genome-Phenome Archive” (#441914366) and from the European Union’s Horizon Europe research and innovation program via the project ERDERA under grant agreement no. 101156595. The TUM IT infrastructure was co-funded by the 10.13039/501100001659DFG, project ID 461264291.

## Author contributions

Conceptualization, H.M., J.K., and R.M.; data curation, H.M., H.K., G.J., D.M., and J.W.U.; formal analysis, H.M., B.A., V.A.Y., J.G., and J.V.; methodology, H.M., H.K., G.J., E.S., and A.S.; funding acquisition, H.M., H.K., M.S.Z., V.A.Y., J.G., J.W.U., H. Houlden, and J.K.; investigation, all authors; recruitment and clinical and diagnostic evaluations, H.M., M.Z., I.A., R.S.A., K.B., S.E., H. Hussein, M.M.N., H.M.E., J.R.A., T.I.O., M.A., E.A., T.S., F.S.A., and H. Houlden; supervision, M.R.R., F.S.A., J.G.G., J.W.U., H. Houlden, J.K., and R.M.; writing – original draft, H.M., H.K., G.J., M.S., J.K., and R.M.; writing – review & editing, all authors.

## Declaration of interests

V.A.Y. is founder, shareholder, and managing director of OmicsDiscoveries GmbH.

## Web resources

gnomAD, https://gnomad.broadinstitute.org/
GTEX, https://www.gtexportal.org/home/
OMIM, https://www.omim.org/

## Supplemental information

Document S1. Figures S1–S11 and Tables S2–S11

Table S1. Detailed phenotype of affected individuals with homozygous MDGA2-related DEE

Document S2. Article plus supplemental information
